# A Young Man Presenting with Pleuritic Chest Pain and Fever after Electrophysiological Study and Implantable Cardioverter-Defibrillator Placement: Diagnostic Difficulties and Value of Bedside Thoracic Sonography

**DOI:** 10.1155/2015/801328

**Published:** 2015-10-21

**Authors:** Antonio Faraone, Alberto Fortini

**Affiliations:** Department of Internal Medicine, “San Giovanni di Dio” Hospital, Via di Torregalli 3, 50143 Florence, Italy

## Abstract

We describe the case of a 23-year-old man presenting with recurrent pleuritic chest pain and prolonged fever after electrophysiology testing and placement of an implantable cardioverter-defibrillator because of a suspected arrhythmogenic right ventricular dysplasia. The clinical suspicion was initially directed toward pneumonia with pleural effusion and later toward an infection of the cardiac device complicated by septic pulmonary embolism. The definitive diagnosis of pulmonary embolism and infarction was suggested by a point-of-care thoracic sonography, performed at the bedside by a clinician caring for the patient, and then confirmed by contrast enhanced computed tomography, which also showed thrombosis of the left iliofemoral vein, site of percutaneous puncture for cardiac catheterization. Prolonged fever was attributable to a concomitant Epstein-Barr virus primary infection that acted as confounding factor. The present report confirms the value of bedside thoracic sonography in the diagnostic evaluation of patients with nonspecific respiratory symptoms.

## 1. Introduction

Venous thrombosis and pulmonary embolism (PE) are rare complications of femoral venous catheterization in patients undergoing electrophysiological procedures [1, 2]. Symptoms of PE are nonspecific, and a high degree of clinical suspicion is necessary to diagnose this potentially fatal disorder. Thoracic ultrasound (US) is an imaging technique easily accessible for clinicians directly at the point-of-care and has been shown to be a valuable tool in the diagnostic work-up of patients with respiratory symptoms and clinically suspected PE [3–6]. We describe the case of a 23-year-old man, who developed iliofemoral vein thrombosis and PE after electrophysiology testing and placement of an implantable cardioverter-defibrillator (ICD). The unusual clinical presentation, further confounded by a concomitant primary Epstein-Barr virus (EBV) infection, posed some diagnostic difficulties. PE and infarction were suggested by bedside thoracic US and later confirmed by contrast enhanced computed tomography (CT) scan.

## 2. Case Presentation

A 23-year-old man, suspected of having arrhythmogenic right ventricular dysplasia, underwent right heart catheterization for electrophysiology testing, via left femoral vein approach, and placement of an ICD, through the left subclavian vein. Five days later, he presented to the emergency department (ED) of our institution because of left pleuritic chest pain. On examination, he showed mild tachypnea, normal body temperature, and slightly diminished breath sounds over the left lung base. Oxygen saturation was 98% on ambient air. Electrocardiogram confirmed the presence of a known incomplete right bundle brunch block with T-wave inversions in leads V1–V3, in the absence of other remarkable findings, except for heart rate of 100 beats per minute. Chest radiograph revealed mild left pleural effusion. The patient was discharged with diagnosis of pleurisy and a seven-day amoxicillin clavulanate treatment was prescribed.

Twelve days later, the patient newly presented to the hospital and was admitted to our medical ward, because of a 3-day history of right pleuritic chest pain, cough, and high-grade fever. His physical examination showed body temperature of 38.4°C, arterial blood pressure of 105/70 mmHg, heart rate of 105 beats per minute, respiratory rate of 20 breaths per minute, right basal hypophonesis on chest auscultation, no heart murmurs, no leg swelling, no enlarged lymph nodes, and no local signs of ICD pocket infection. A new chest radiograph documented bilateral mild pleural effusion and consolidation at the right lung base. Transthoracic echocardiogram (TTE) showed no cardiac dysfunction and excluded recognizable endocardial or ICD leads vegetation. Laboratory tests revealed a normal white blood cells count, with lymphocytosis and presence of atypical lymphocytes. C-reactive protein level was high (16.4 mg/dL), and procalcitonin was normal. Other test results are shown in Table 1. Diagnosis of pneumonia and consensual pleural effusion was proposed and after drawing blood cultures, the patient was started on ceftriaxone and azithromycin.

**Figure 1:**
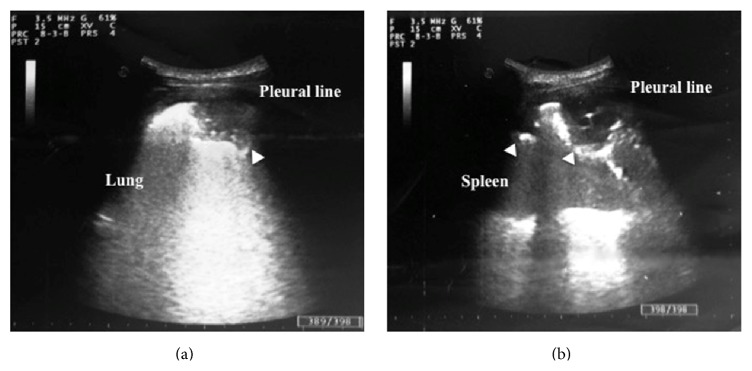
Chest sonography (3.5 MHz convex scanner): hypoechoic, wedge, and rounded shaped, subpleural consolidations (arrowheads) in the right (a) and left lung (b).

On the third hospital day, the patient's clinical picture was unchanged, despite antibiotic treatment. Absence of clinical improvement and persistent atypical lymphocytosis triggered a point-of-care thoracoabdominal ultrasound examination, which was conducted at the bedside by one of the internal medicine physicians caring for the patient, using a MyLab 40 US portable system (Esaote) equipped with a 3.5 MHz convex transducer. Abdominal examination detected splenomegaly (craniocaudal length of 14 cm). Chest sonography showed mild bilateral pleural effusion and pleurally based, hypoechoic, wedge, and rounded shaped lung consolidations (2 in the lower left lobe and 1 in the lower right lobe, maximum size 50 mm) presenting scant or absent bronchoaerogram (Figure 1). Thoracic findings were suggestive of PE and infarction; nevertheless, the association of multiple lung consolidations, fever and other sepsis signs, and history of ICD placement oriented diagnostic suspicion toward a SPE complicating ICD leads infection. With the aim of a more sensitive assessment of endocardial and ICD leads involvement, a transesophageal echocardiogram (TEE) was ordered, which was refused by the patient. Empiric vancomycin treatment was added to cover methicillin-resistant staphylococci. A chest contrast enhanced CT scan documented filling defects within pulmonary arteries for posterior basal segments of both lungs and confirmed the presence of basal subpleural consolidations compatible with infarct lesions. No reliable images of lung abscess were detected. Furthermore, an abdominal CT scan revealed thrombosis of the left iliofemoral vein, site of recent percutaneous puncture for cardiac catheterization. A definitive diagnosis of PE complicating iliofemoral vein thrombosis was formulated, and the patient was started on rivaroxaban. Considering the association of atypical lymphocytosis, splenomegaly, and prolonged fever, a concomitant viral illness was suspected; serologic testing confirmed an EBV primary infection (Table 2). All blood cultures drawn during hospital stay (6 sets) were negative, and procalcitonin remained persistently normal. In the absence of reliable imaging and laboratory findings of bacterial infection, antibiotics were withheld on day 7 after admission. Pleuritic chest pain and fever disappeared on day 9, and the patient was discharged home. Ambulatory controls in the following weeks showed excellent clinical conditions and progressive normalization of laboratory tests. Rivaroxaban was stopped after 3 months and the patient had an uneventful course.

## 3. Discussion

Deep vein thrombosis (DVT) and PE are very rare complications of right heart catheterization in patients undergoing electrophysiological procedures [1]. In a recent study by Alizadeh et al., none of the 200 enrolled patients developed DVT after right-sided electrophysiological procedures via the femoral vein approach [2].

PE manifestations are nonspecific; therefore a high index of clinical suspicion is required for prompt diagnosis and treatment [3]. Pleuritic chest pain is a possible presenting symptom of PE: 5 to 20 percent of patients who present to the ED with pleuritic pain are diagnosed with this potentially lethal disorder [7]. In a study by Pollack et al., pleuritic chest pain was reported in 39% of patients with confirmed PE; fever was present in only 10% of patients [8].

CT pulmonary angiography (CTPA) is the first-line imaging method for assessing patients with clinically suspected PE [9]. Thoracic sonography represents a safe and effective complementary tool for the diagnosis of PE, which offers the opportunity of a point-of-care assessment of patients with clinically suspected PE and appears suitable for the differential diagnosis of patients with nonspecific respiratory symptoms [3–6]. Ultrasound detects alterations in the pleura and lung parenchyma associated with thromboembolism. Typical findings are pleural effusions, which can be localized or basal, and wedge, rounded, or polygonal shaped, multiple hypoechoic subpleural parenchymal lesions, corresponding to peripheral lung infarctions. Most of PE-related lesions are localized in the dorsobasal segments of the lung and present variable size, from 5 mm up to several centimeters [3, 4]. Accuracy of thoracic US for the diagnosis of PE is high. A recent meta-analysis by Squizzato et al. reported overall sensitivity of 87.0% and specificity of 81.8% [5]. Concurrent TTE and compression ultrasound of the leg veins improve the diagnostic accuracy of sonographic procedures [10]. Chest sonography can substitute CTPA in patients with hemodynamic instability or contraindication to CTPA. However, a negative chest US result does not rule out a PE and, in case of high clinical suspicion, further diagnostic evaluation is needed [3–5].

Infection is an uncommon but serious complication of implantable cardiac electronic device (ICED) placement, which can manifest as infection of the generator pocket or of the leads, and can involve endocardial structures. As the prevalence of patients with these devices is increasing, the incidence of ICED related infections is rising. ICED infections now constitute about 10% of all endocarditis. Staphylococci cause the majority of infections [11]. The interval between ICED placement and the onset of infection is widely variable, from days to years [12]. Diagnosis is based on clinical history, microbiological tests, echocardiography, and other complementary imaging modalities (chest radiograph, CT scanning, and CTPA). The Duke criteria can be used to assist the diagnostic process. TEE has a higher diagnostic sensitivity than TTE; several studies have demonstrated TEE identification of lead involvement in 90–96% of cases, compared with 22–43% with TTE [11].

SPE, considered as minor Duke criterion, is a possible manifestation of ICED infection. It generally presents with an insidious onset of fever, respiratory symptoms, and lung infiltrates [13]. In SPE, the embolic blood clot that leads to an infarction in the pulmonary vasculature also contains microorganisms that incite a focal abscess. Parenchymal lesions are usually multiple, wedge shaped, or nodular, with a peripheral distribution and a tendency for cavitation. Suspected SPE must trigger prompt diagnostic evaluation for identification of intracardiac infection [13].

In conclusion, our patient presented with nonspecific respiratory manifestations and possible sepsis signs, apparently determined by pneumonia and consensual pleurisy. Bedside thoracic US detected pleural and lung alterations characteristic of PE and infarction; however, in light of clinical context, a differential diagnosis of SPE complicating an ICD related infection was considered. TTE and blood cultures did not corroborate this hypothesis, and CT scan definitively diagnosed PE originating from left iliofemoral vein thrombosis. The intercurrent infectious mononucleosis, responsible for atypical lymphocytosis, splenomegaly, and, probably, prolonged fever, acted as confounding factor.

Thoracic US confirmed being a valuable real-time diagnostic tool in the hands of a skilled clinician, which can efficiently supplement clinical examination of patients with respiratory symptoms directly at the bedside. The present report supplies further evidence that point-of-care sonography should be part of the modern internist expertise.

## Figures and Tables

**Table 1:** Laboratory data.

Variable	Admission	Reference range
Arterial pH	7.42	7.35–7.45
Arterial pCO_2_, mmHg	40	35–45
Arterial pO_2_, mmHg	74	80–100
Lactate, mmol/L	0.6	0.5–2.2
Hematocrit, %	34.1	39.0–50.0
Hemoglobin, g/dL	11.3	13.2–17.0
White blood cell count, ×10^9^/L	8.33	4.4–10.1
Differential count, %		
Neutrophils	26.4	40–80
Lymphocytes	56.1	20–40
Monocytes	15.7	2–10
Eosinophils	0.6	0.0–6.0
Platelet count, ×10^9^/L	182	150–400
Blood urea nitrogen, mg/dL	31	10–50
Creatinine, mg/dL	1.1	0.7–1.2
Creatine kinase, U/L	42	38–174

**Table 2:** Laboratory data.

Variable	Day 5 after admission	Reference range
EBV VCA IgG, AU/mL	76.30	Negative <20.00 Positive >20.00

EBV VCA IgM, AU/mL	>160	Negative <20.00 Positive >40.00

EBV EA IgG, U/mL	72.00	Negative <10.00 Positive >40.00

EBV EBNA IgG, AU/mL	3.40	Negative <5.00 Positive >20.00

Cytomegalovirus IgG, AU/mL	0.10	Negative <6.0 Positive >6.0

Cytomegalovirus IgM, S/CO	0.81	Negative <0.85 Positive >1.0
